# Supplementary material for: Inhibition of host PARP1 contributes to the anti-inflammatory and antitubercular activity of pyrazinamide
Source: Nat Commun. 2023 Dec 9;14:8161. doi: 10.1038/s41467-023-43937-1 (PMC10710439; doi:10.1038/s41467-023-43937-1)

Inhibition of host PARP1 contributes to the anti-inflammatory and antitubercular activity of pyrazinamide

Stefanie Krug[1,2], Manish Gupta[1,2], Pankaj Kumar[1,2], Laine Feller[2], Elizabeth A. Ihms[1,2], Bong Gu Kang[3,4], Geetha Srikrishna[1,2], Ted M. Dawson[3,4,5,6], Valina L. Dawson[3,4,5,7], William R. Bishai[1,2]*

SUPPLEMENTARY FIGURES

Supplementary Table 1: Antimycobacterial activity of Talazoparib (Tp).

	MIC (µg/ml)	Maximum *M.tb* inhibition	
		Concentration	% Inhibition
INH	0.08	2.56 µg/ml	98.4 (98.3 – 98.5)
PZA	-	128 µg/ml	8.8 (3.8 – 9.2)
Tp	-	128 µg/ml	48.2 (34 – 68.2)
DMSO	-	3.38 %	24 (19.6 – 81)

MICs, defined as the minimal concentration resulting in >90% growth inhibition of *M.tb* H37Rv, were determined by Alamar Blue assay.
Data represent the average and range of three independent experiments.
INH, isoniazid. PZA, pyrazinamide.

Supplementary Table 2: CFU progression in WT and PARP1$^{-/-}$ mice with and without PZA treatment.

Time (months)*	Treatment	WT			PARP1$^{-/-}$			*P*
		Mean	SEM	n	Mean	SEM	n	
-1	-	1.90	0.08	11	1.88	0.06	11	0.79
0	-	7.24	0.09	6	6.95	0.24	6	0.998
2	-	5.87	0.08	5	6.21	0.17	6	0.99
	PZA	4.44	0.21	8	5.23	0.23	8	*0.0493*

129S1 (WT) and PARP1$^{-/-}$ mice were infected with *M.tb* H37Rv and treated as outlined in Figure 6 A. Statistical differences were assessed by 2way ANOVA with Šidák’s multiple comparisons test.
*Time in months relative to treatment initiation.

Supplementary Table 3: Real-time PCR primer sequences.		
Gene	Forward primer (5’ → 3’)	Reverse primer (5’ → 3’)
Gapdh	AACGGATTTGGCCGTATTGG	CATTCTCGGCCTTGACTGTG
Tnfa	CCAACGGCATGGATCTCAAA	CCCTTGAAGAGAACCTGGGA
iNOS	ATGCGAAAGGTCATGGCTTC	CCCAAATGTGCTTGTCACCA
Cxcr5	ACACTGCTGTCTCAATCCCA	AAGGTGGTGAGGGAAGTAGC
Mcp-1 (Ccl2)	AACTGCATCTGCCCTAAGGT	CTGTCACACTGGTCACTCCT
Ifnb	ATGAACTCCACCAGCAGACA	ATCCAGGCGTAGCTGTTGTA
Ifit1	TCCGTAGGAAACATCGCGTA	CACATTGTCCTGCCTTCTGG
Ifit3	CTATCACATGGGCCGTCTCT	TCTGGGCATTCCATGCTGTA

Supplementary Fig. 1

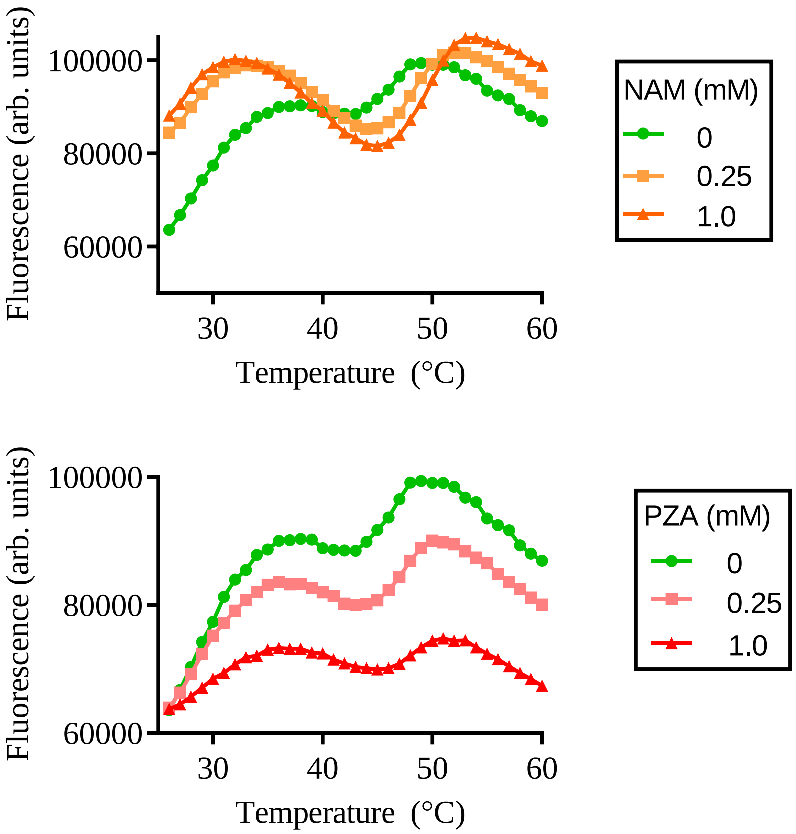

Supplementary Fig. 1: PARP1 fluorescence vs. temperature plots.

Fluorescence vs. temperature plots of PARP1 (green) in the presence of 0.25 mM or 1.0 mM NAM (orange) or PZA (red). Each data point indicates SYPRO orange fluorescence intensity at the corresponding temperatures.

Supplementary Fig. 2

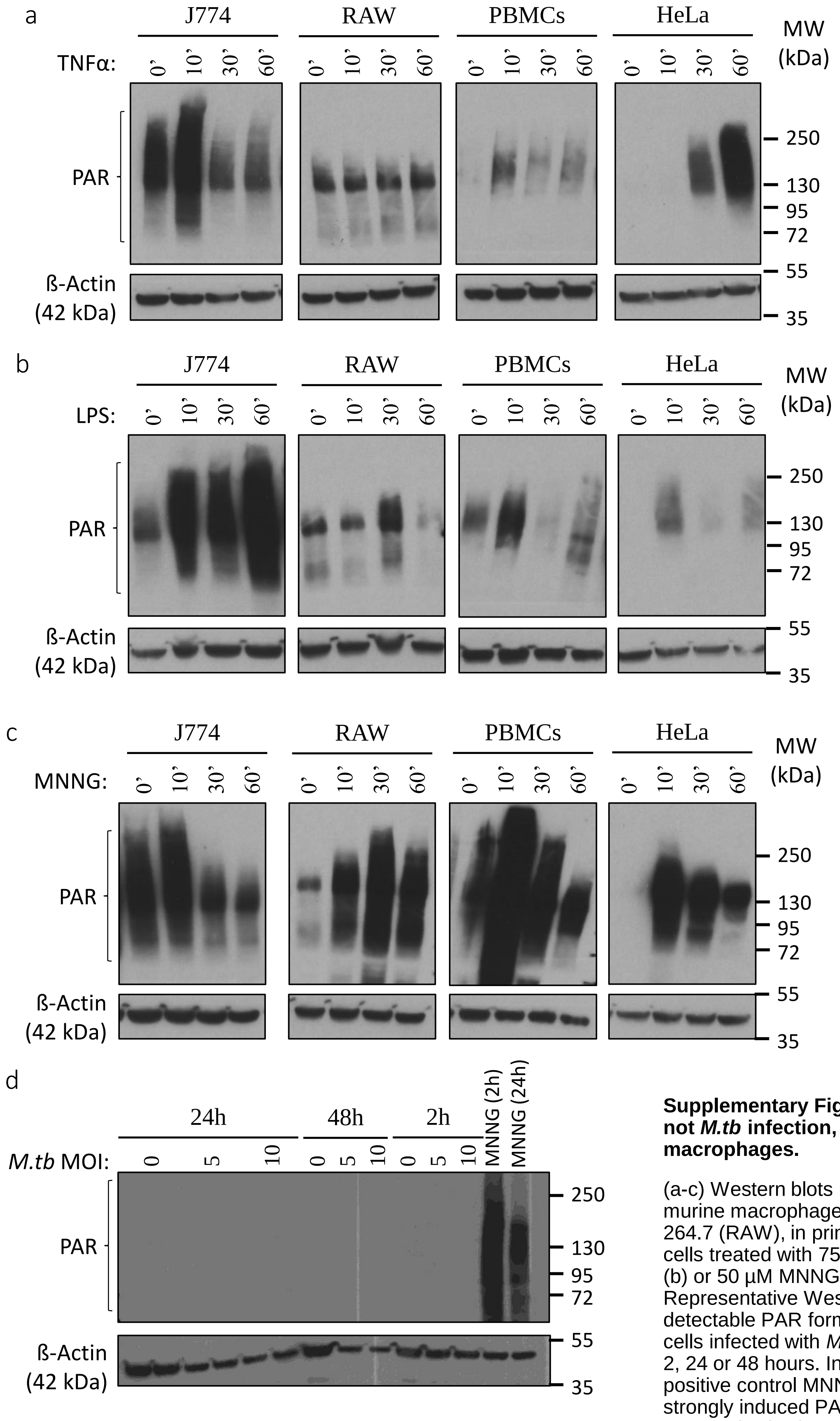

Supplementary Fig. 2: TNFα or bacterial LPS, but not *M.tb* infection, induce PAR formation in macrophages.

(a-c) Western blots illustrating PAR formation in the murine macrophage lines J774A.1 (J774) and RAW 264.7 (RAW), in primary human PBMCs, and in HeLa cells treated with 75 ng/ml TNFα (a), 500 ng/ml LPS (b) or 50 µM MNNG (c) for up to 60 minutes. (d) Representative Western blot showing absence of detectable PAR formation in PMA-differentiated THP1 cells infected with *M.tb* H37Rv at an MOI of 5 or 10 for 2, 24 or 48 hours. In contrast, treatment with the positive control MNNG (50 µM) for 2 or 24 hours strongly induced PAR formation. Experiments were repeated 2 (a-c) or 4 (d) times with similar results.

Supplementary Fig. 3

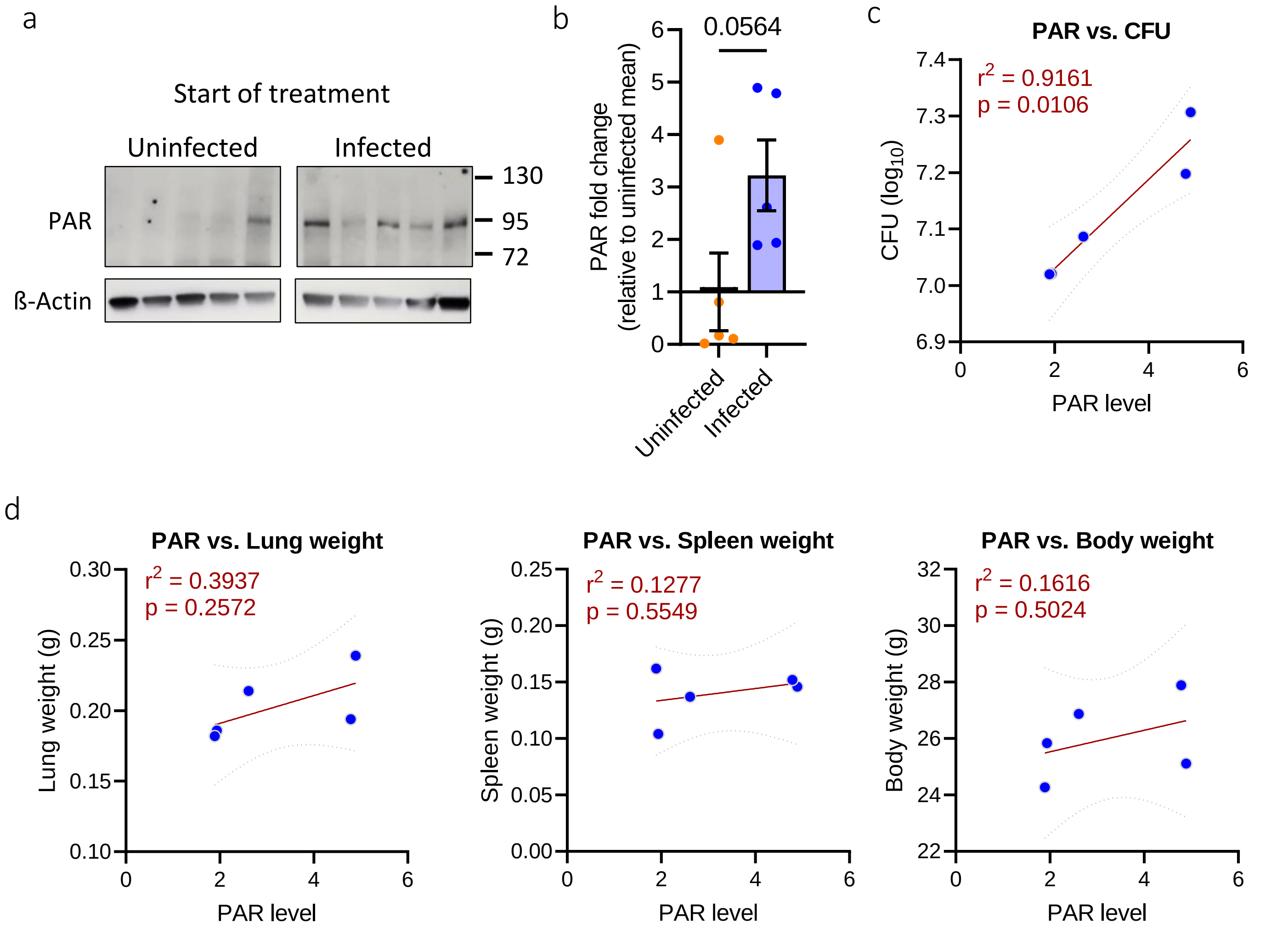

Supplementary Fig. 3: TB infection induces lung PAR formation that correlates with bacterial burden.

(a) Representative Western blots showing PAR levels (top) or the loading control β-Actin (bottom) in mouse lungs one month after infection (at the start of treatment) or in age-matched uninfected mice. Each lane represents an individual mouse (n=5). (b) Densitometric analysis showing the change in ß-Actin-normalized lung PAR intensity at the start of treatment in infected compared to age-matched uninfected mice (n=5). Each symbol represents an individual mouse, and bars the mean ± SEM. Statistical difference was assessed by unpaired t-test. (c-d) Simple linear regression analyses of lung PAR levels (fold change relative to uninfected mean) and the corresponding lung bacterial burden (in $\log_{10}$ CFU; c), lung, spleen or body weights (in grams; d) at the start of treatment. The goodness-of-fit (r^2) of the best-fit linear regression line (red), 95% confidence intervals (dotted lines) and probability values are indicated in each plot. The increase in PAR formation in response to *M.tb* infection correlates strongly with the bacterial burden but not lung, spleen or body weight of mice.

Supplementary Fig. 4

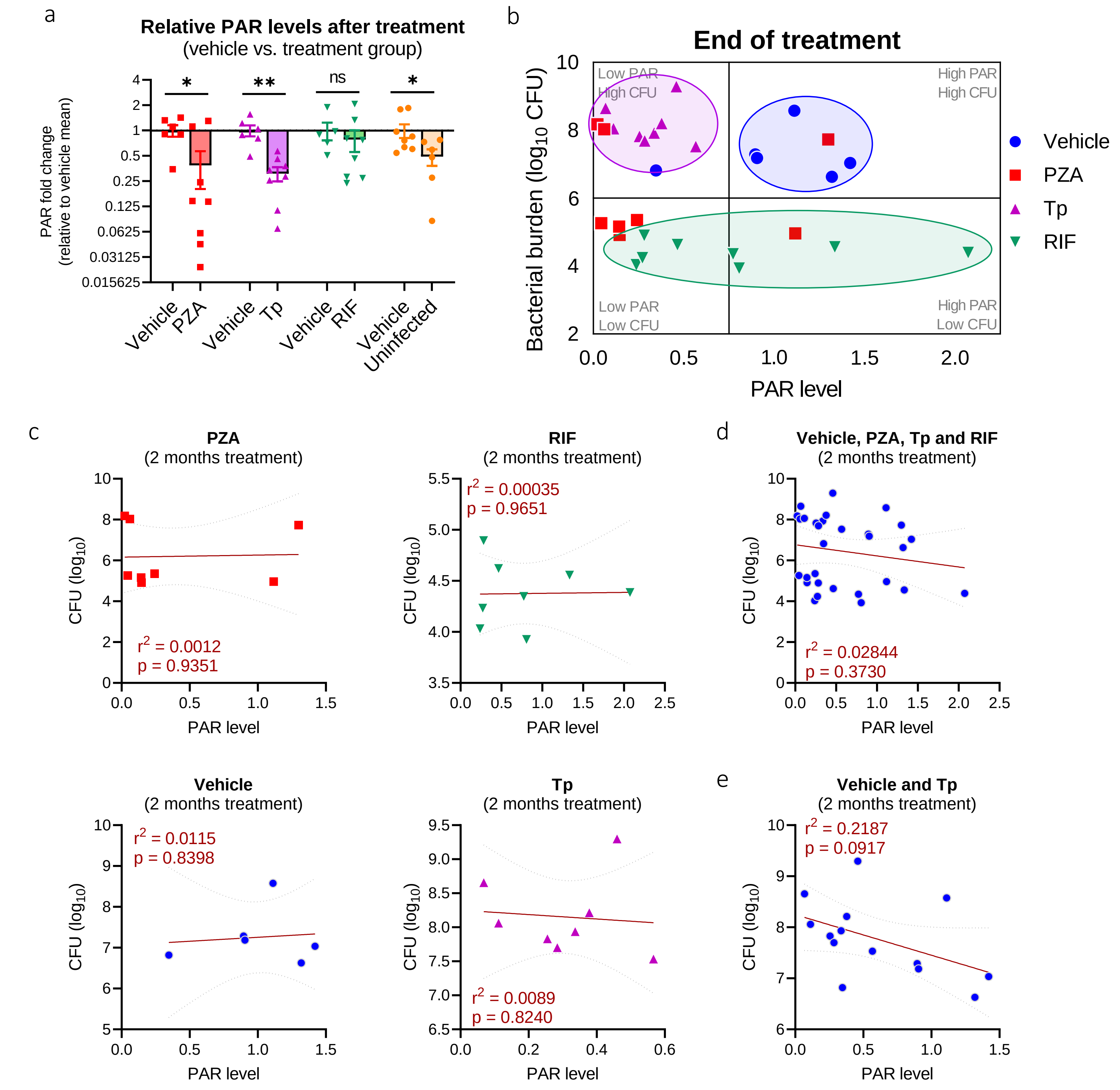

Supplementary Fig. 4: PARP inhibition without effective antibiotics may impair chronic TB containment.

(a) ß-Actin-normalized lung PAR levels in corresponding vehicle-treated mice alongside PZA-, Tp- or RIF-treated or uninfected mice shown in figure 3c. Each symbol represents an individual mouse, and bars the group mean ± SEM (n= 5 (Veh, vs. RIF), 6 (Veh, vs. PZA or Tp; Uninfected) or 8 (all other)). ns, not significant; *, $p < 0.05$; **, $p < 0.01$, versus vehicle by 2-way ANOVA with uncorrected Fisher's least significant difference (LSD) test. (b) Scatter plot showing lung PAR level (x) and corresponding bacterial burden (y) for each vehicle-, Tp-, PZA- or RIF-treated mouse after 2 months of treatment (n=6 (vehicle) or 8 (all other)). Four quadrants, based on clustering of vehicle-treated mice, visually indicate mice with reduced bacterial burden (<6 log_{10} CFU; below horizontal line) and/or reduced PAR levels (<0.75 relative to vehicle mean; left of vertical line). RIF-treated mice (green oval) have uniformly low CFU but variable PAR levels, while Tp-treated mice (purple oval) have uniformly high CFU but low PAR. In most PZA-treated mice, bacterial killing coincided with PARP inhibition (lower left quadrant). (c-e) Simple linear regression analyses of lung PAR levels (fold change relative to vehicle mean) and the corresponding lung bacterial burden (in log_{10} CFU) after 2 months of treatment with PZA, RIF, vehicle or Tp (c), for all treatment groups combined (d), or for all mice that did not receive antibiotics (e) (n=6 (vehicle) or 8 (all other)). The goodness-of-fit (r^2) of the best-fit linear regression line (red), 95% confidence intervals (dotted lines) and probability values are indicated in each plot. Without antibiotics, PAR levels and bacterial burden are trending toward a negative correlation in the chronic phase of infection.

Supplementary Fig. 5

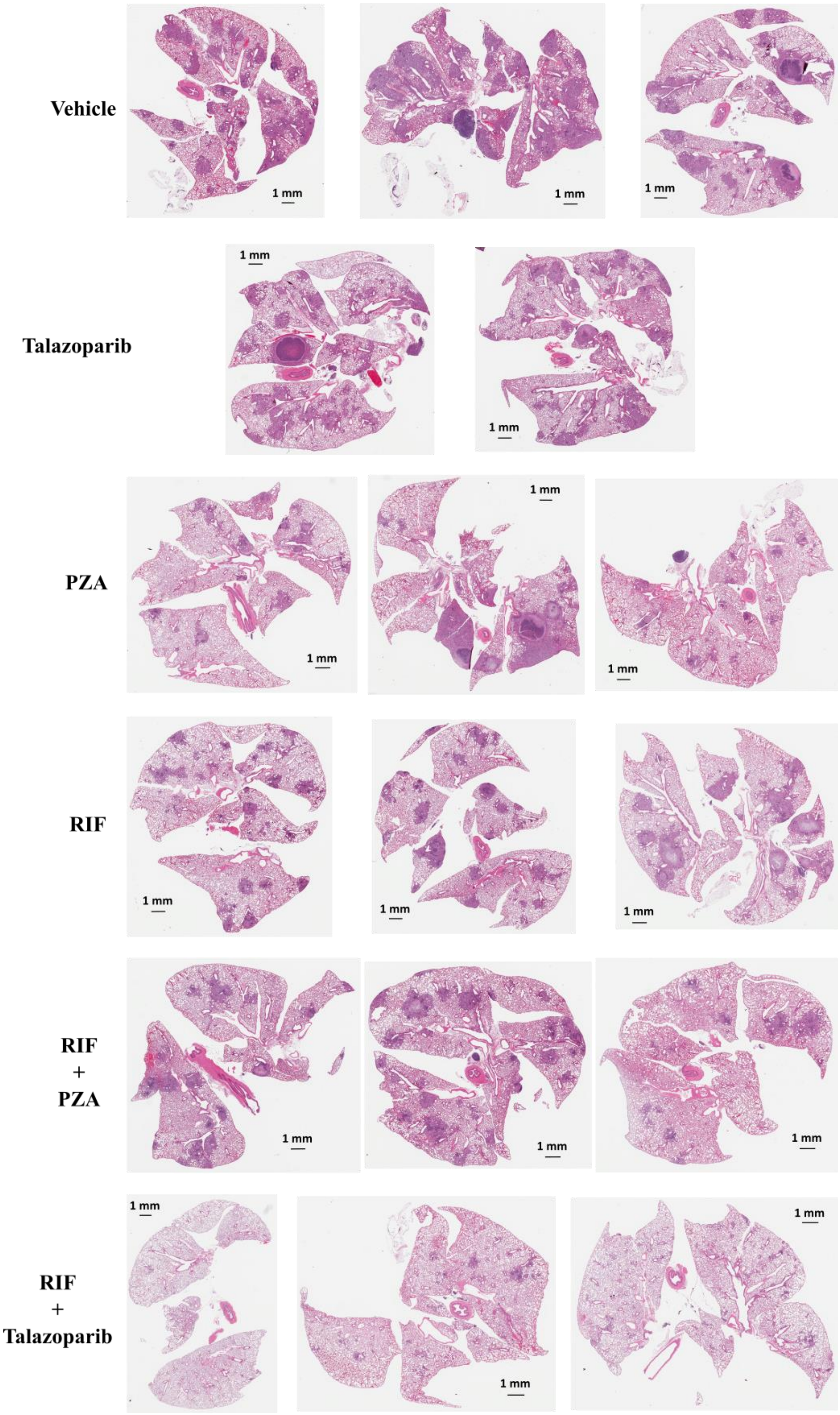

Supplementary Fig. 5: Adjunctive PARP inhibition reduces lung pathology in chronic TB infection.

Lungs of chronically *M.tb*-infected C3HeB/FeJ mice after receiving vehicle, PZA or the PARP inhibitor talazoparib with or without RIF for 8 weeks. Lungs were formalin-fixed, sectioned, stained with hematoxylin and eosin (H&E) and digitally scanned at 40x. Each section represents an individual mouse (n=2 (Talazoparib) or 3 (all other groups)). A repository of the digitally scanned, H&E-stained lung sections used for histology analysis is publicly available at https://tinyurl.com/4az6ueks. Analysis was done blinded to the treatment group. While lungs of mice treated with talazoparib alone looked similar to those of vehicle-treated mice, talazoparib dramatically decreased lung pathology when given in combination with RIF despite the increased lung bacterial burden compared to RIF alone.

Supplementary Fig. 6

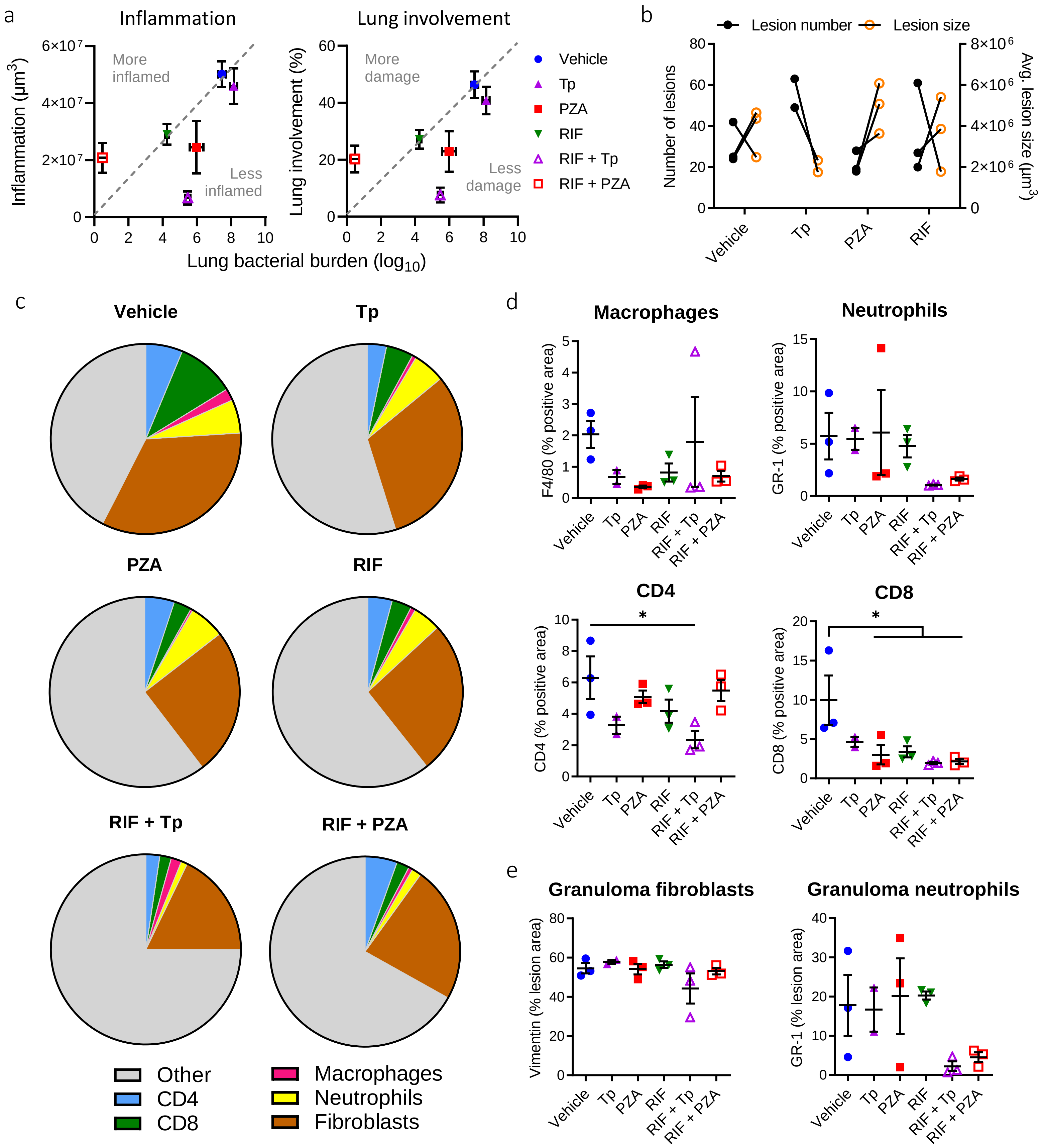

Supplementary Fig. 6

Supplementary Fig. 6: Influence of PZA and Tp on pathology and immune cell composition of *M.tb.*-infected mouse lungs.

Lung histopathology and cellular composition of female *Mtb*-infected C3HeB/FeJ mice (implantation: 111 ± 9 CFU) 3 months post infection following 2 months of treatment with PZA (150 mg/kg), talazoparib (Tp; 0.5 mg/kg) or vehicle (1.97% DMSO in 0.5% CMC), alone or in combination with RIF (10 mg/kg). (n=2 (Tp) or 3 (all other groups)). (a) Mean lung inflammation or % lung involvement, plotted against the corresponding mean lung bacterial burden for each treatment group. Each symbol indicates the group mean ± SEM. The dotted line indicates the expected inflammation or consolidation corresponding to bacterial burden without anti-inflammatory treatments (i.e., Tp, PZA) based on the correlates of vehicle and RIF monotherapy. PZA and Tp lower inflammation and lung damage more than predicted by CFU alone. (b) Pairwise comparison of lesion number and mean lesion size (connecting lines indicate corresponding values). Every pair represents an individual mouse. (c-e) Macrophages (F4/80), neutrophils (GR-1), CD4+ T cells (CD4), CD8+ T cells (CD8) and fibroblasts (vimentin) were immunohistochemically labeled in formalin-fixed lung sections. The areas that stained positive for each marker were quantified by a veterinary pathologist blinded to experimental design and are expressed as a percentage of the total lung tissue in each section. (c) Pie charts depicting the mean cellular lung composition at the end of treatment by group. (d) Scatter plots of the corresponding positively stained areas in individual lungs (for fibroblasts, refer to figure 4f). (e) Mean fibroblast (left) and neutrophil (right) staining within 3 independent areas of consolidation (granulomas) per mouse. Foci were defined by vimentin staining. Each symbol represents an individual mouse, with mean ± SEM indicated. Statistical differences between treatment groups and vehicle were determined by one-way ANOVA with Dunnett’s multiple comparisons test. *, $p < 0.05$. Adjunctive PARP inhibition may promote granuloma resolution by reducing T cell, fibroblast and neutrophil infiltration into the lung.

Supplementary Fig. 7

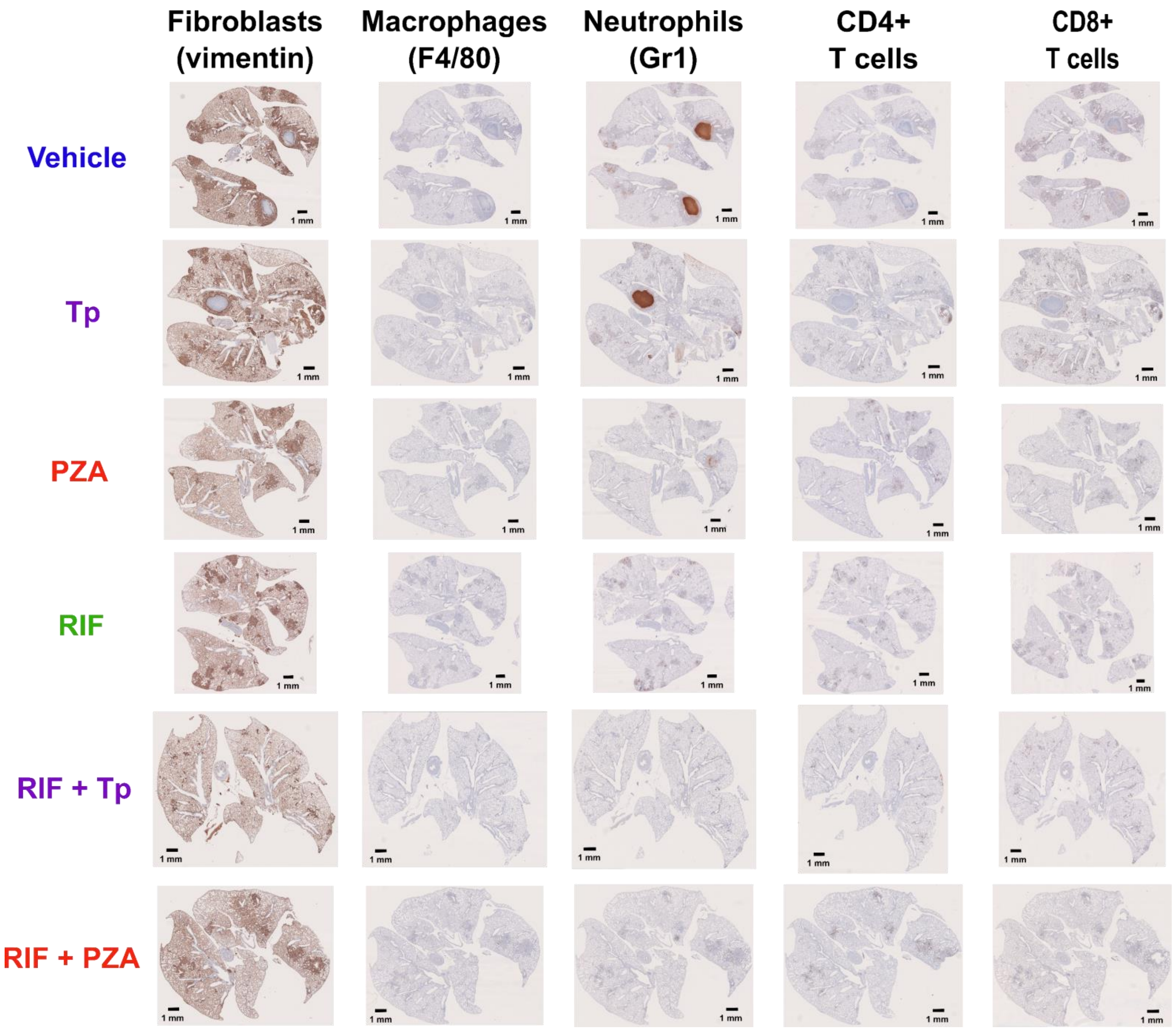

Supplementary Fig. 7: Representative IHC images.

Representative lung histopathology of *Mtb*-infected C3HeB/FeJ mice (implantation: 111 ± 9 CFU) 3 months post infection following 2 months of treatment with PZA (150 mg/kg), talazoparib (Tp; 0.5 mg/kg) or vehicle (1.97% DMSO in 0.5% CMC), alone or in combination with RIF (10 mg/kg). n=2 (Tp) or 3 (all other groups). A repository of the digitally scanned IHC-stained lung sections is publicly available at https://tinyurl.com/3v384esc, and the decoded slide IDs are listed in the Source Data file. Macrophages (F4/80), neutrophils (GR-1), CD4+ T cells (CD4), CD8+ T cells (CD8) and fibroblasts (vimentin) were immunohistochemically labeled in formalin-fixed lung sections. For comprehensive quantification and analysis, see Supplementary figure 6.

Supplementary Fig. 8

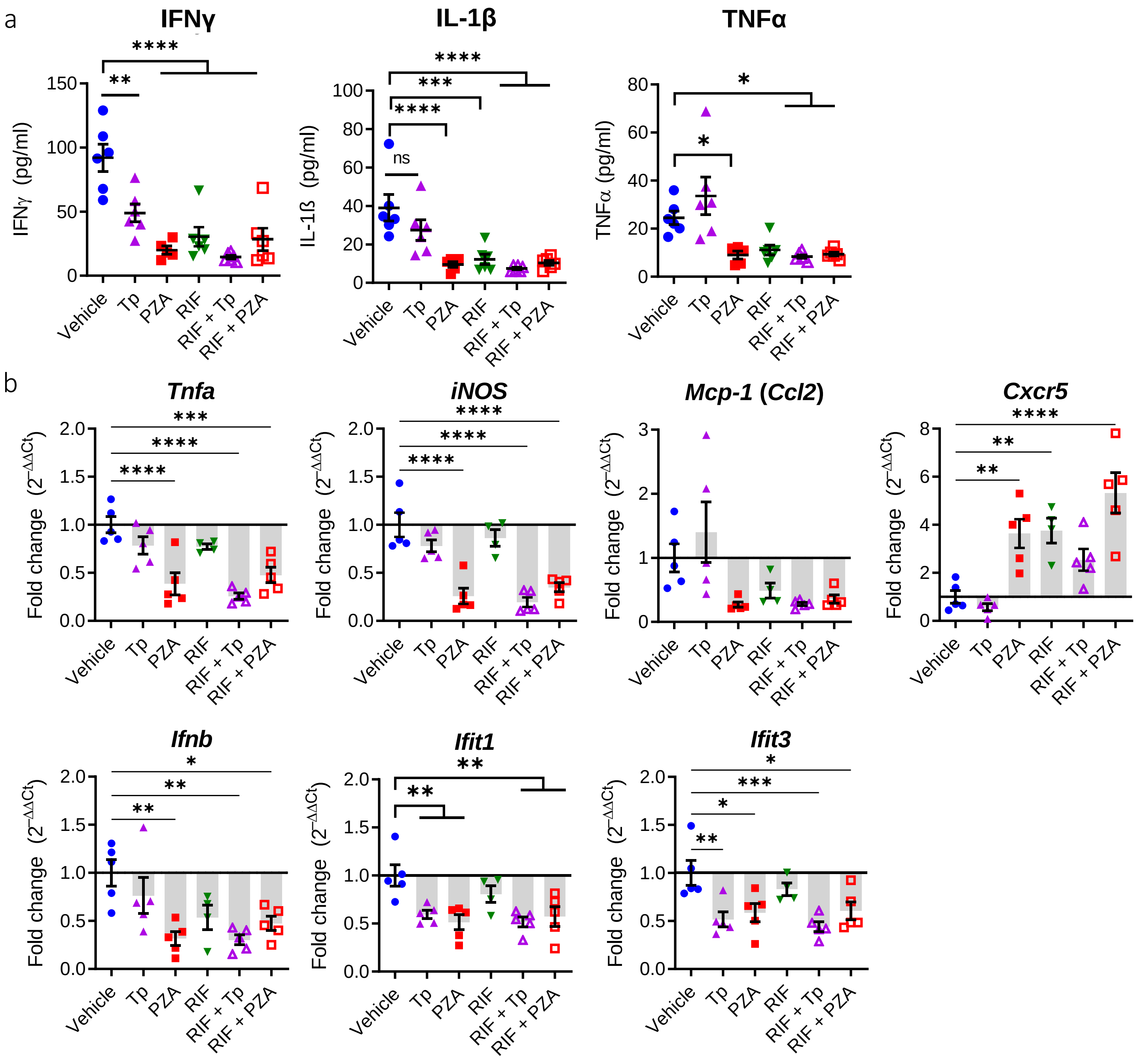

Supplementary Fig. 8: Influence of PZA and Tp on *M.tb.*-induced immune signaling in mouse lungs.

Protein and gene expression analyses of key immune mediators in the lungs of female *Mtb*-infected C3HeB/FeJ mice (implantation: 111 ± 9 CFU) 3 months post infection following 2 months of treatment with PZA (150 mg/kg), talazoparib (Tp; 0.5 mg/kg) or vehicle (1.97% DMSO in 0.5% CMC), alone or in combination with RIF (10 mg/kg). (**a**) Protein levels of lung cytokines quantified by Luminex multiplex assay. Each symbol represents an individual mouse, with mean ± SEM indicated (n = 6). (**b**) Change in cytokine, chemokine or interferon-stimulated gene expression, as determined by quantitative real-time PCR, in mouse lungs after 2 months of treatment relative to the expression in vehicle-treated mice. Each symbol represents an individual mouse, with mean ± SEM indicated (n = 4 (RIF) or 5 (all other groups)). Statistical differences between treatment groups and vehicle were determined by one-way ANOVA with Dunnett's multiple comparisons test. *, $p < 0.05$; **, $p < 0.01$; ***, $p<0.001$; ****, $p<0.0001$; ns, not significant.

Supplementary Fig. 9

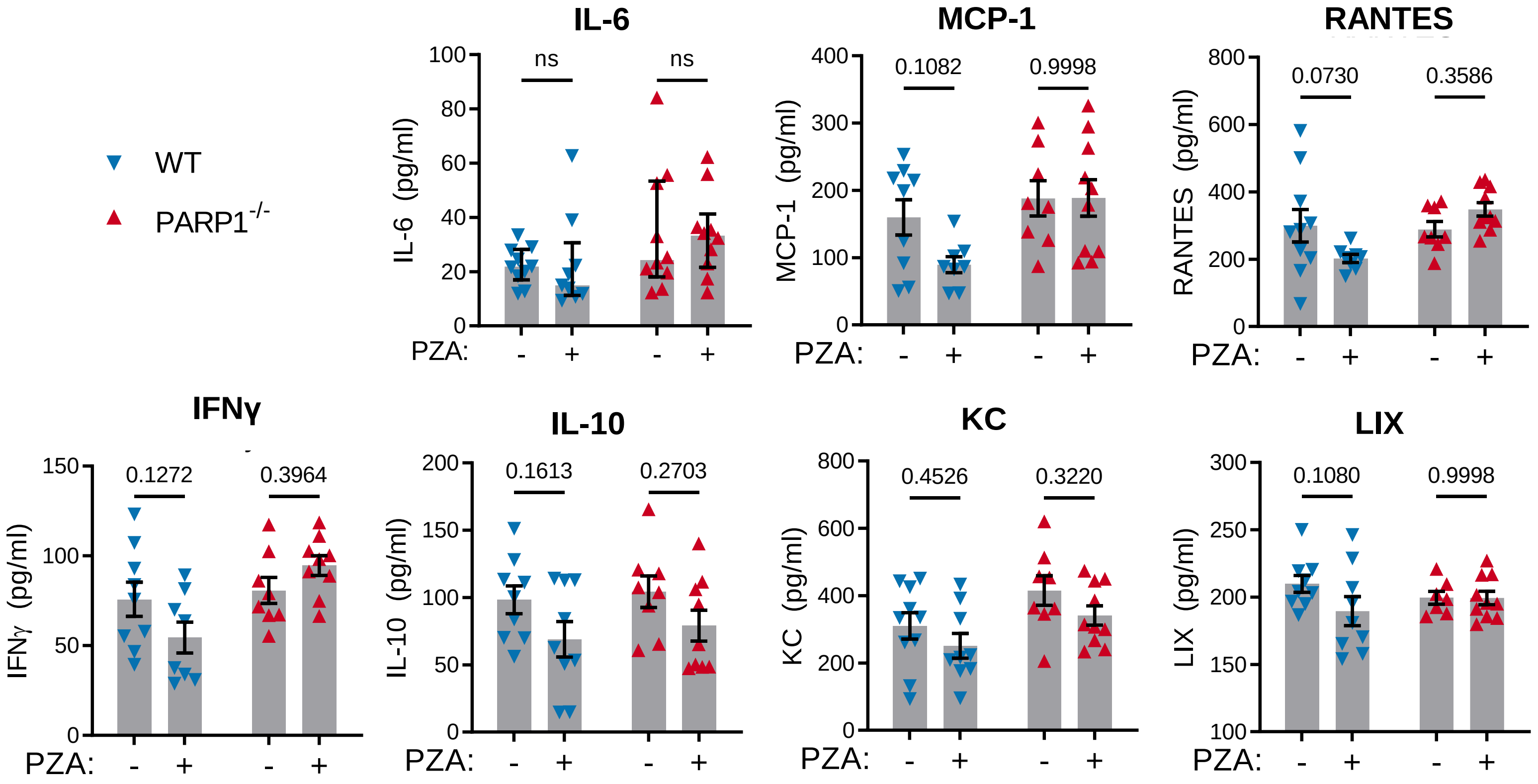

Supplementary Fig. 9: In the absence of bacterial killing, PZA's anti-inflammatory effects are PARP1-dependent.

Male and female PARP1-null (PARP1$^{-/-}$) or 129S1 (WT) mice were aerosol infected with the PZA-resistant *M.tb* H37Rv Δ*pncA* (A146V) mutant (implantation: 54 ± 5 CFU). Starting one month after infection, half of the mice were administered PZA (150 mg/kg) 5 days/week for 2 months. Lung cytokine/chemokine concentrations were quantified by Luminex multiplex assay in untreated (-) or PZA-treated (+) mice at the end of treatment (n=10 (UNT WT, UNT or PZA-treated PARP1$^{-/-}$) or 9 (PZA-treated WT)). Each symbol represents an individual mouse, with median ± interquartile range (IL-6) or mean ± SEM (all other cytokines) indicated. Statistical differences between groups were determined by 2-way ANOVA with Šidák's multiple comparisons test, except for IL-6 which was analyzed by Kruskal-Wallis test with uncorrected Dunn's multiple comparisons test since datapoints were not normally distributed. PZA reduced lung cyto- and chemokine levels in WT but not in PARP1$^{-/-}$ mice.

Supplementary Fig. 10

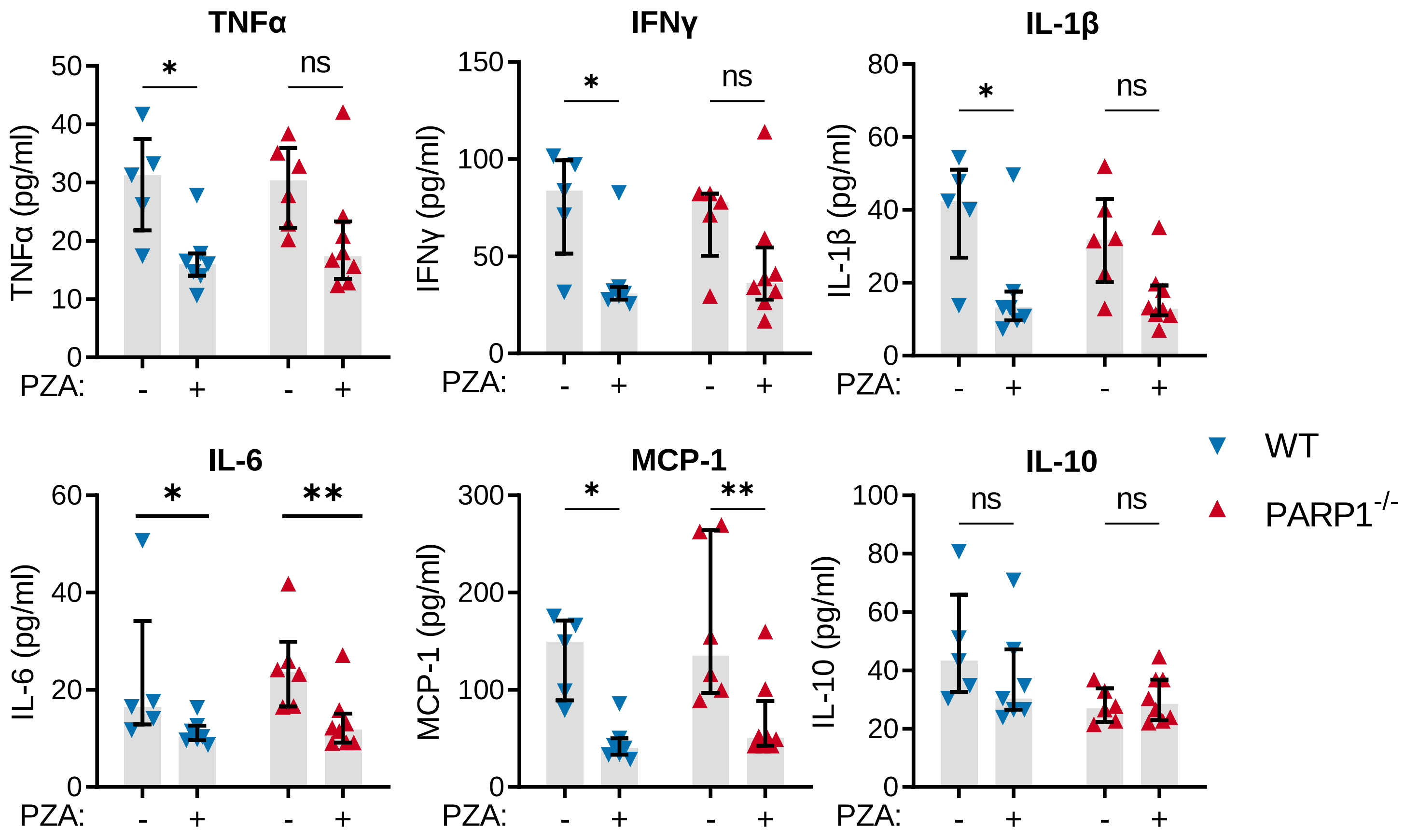

Supplementary Fig. 10: PZA's anti-inflammatory effects are reduced in PARP1-deficient mice infected with PZA-susceptible *M.tb*.

Male and female PARP1-null (PARP1$^{-/-}$) or 129S1 (WT) mice were aerosol infected with PZA-susceptible *M.tb* H37Rv (implantation: 80 ± 7.5 CFU). Starting one month after infection, half of the mice were administered PZA (150 mg/kg) 5 days/week for 2 months. Lung cytokine/chemokine concentrations were quantified by Luminex multiplex assay in untreated (-) or PZA-treated (+) mice at the end of treatment (n=5 (untreated WT), 6 (untreated PARP1$^{-/-}$), 7 (PZA-treated WT) or 8 (PZA-treated PARP1$^{-/-}$)). Each symbol represents an individual mouse, with median ± interquartile range indicated. Statistical differences between groups were determined by Kruskal-Wallis nonparametric test with uncorrected Dunn's multiple comparisons test.

UNCROPPED WESTERN BLOTS

(SUPPLEMENTARY FIGURES)

Supplementary Fig. 2 a-c

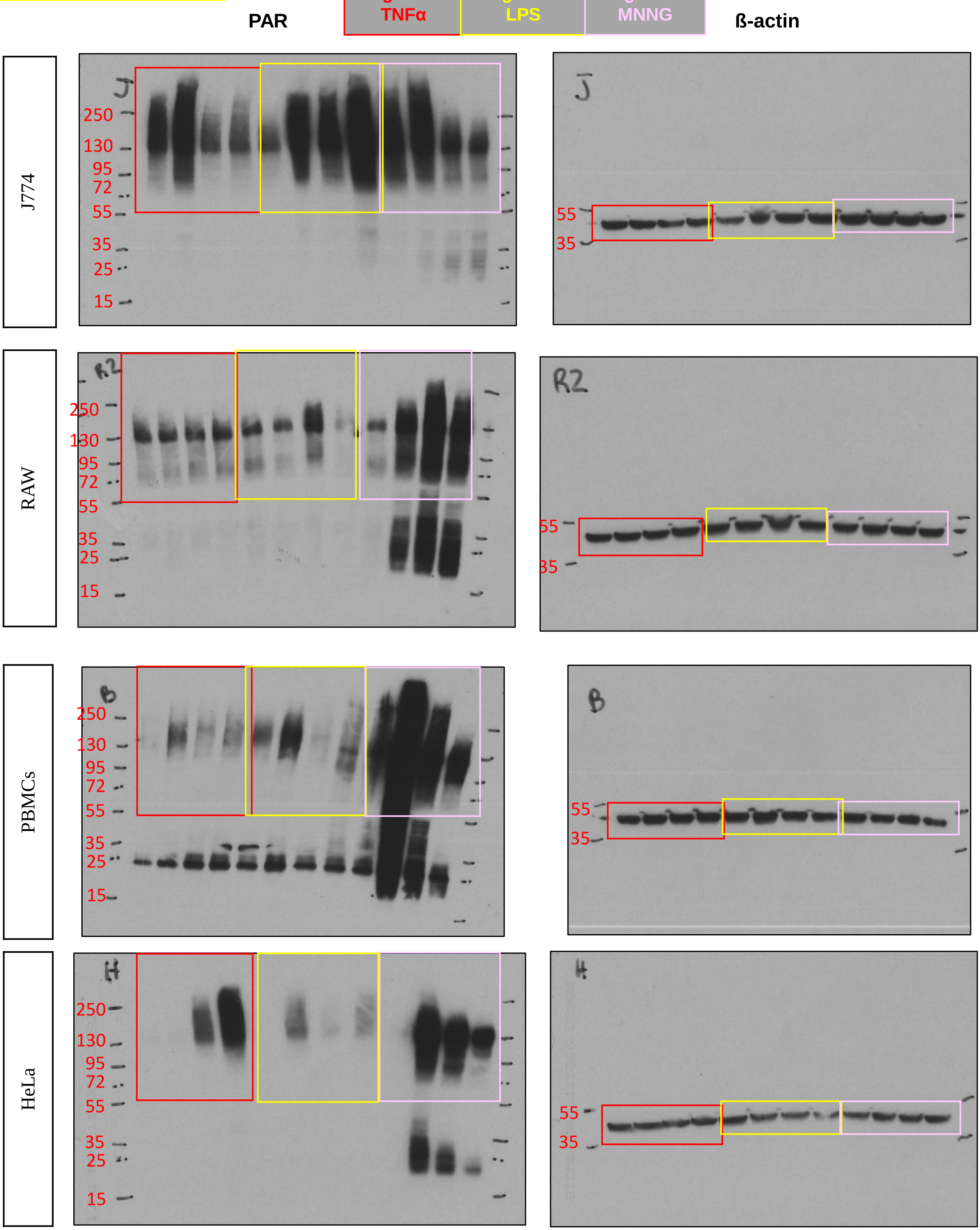

Supplementary Fig. 2a

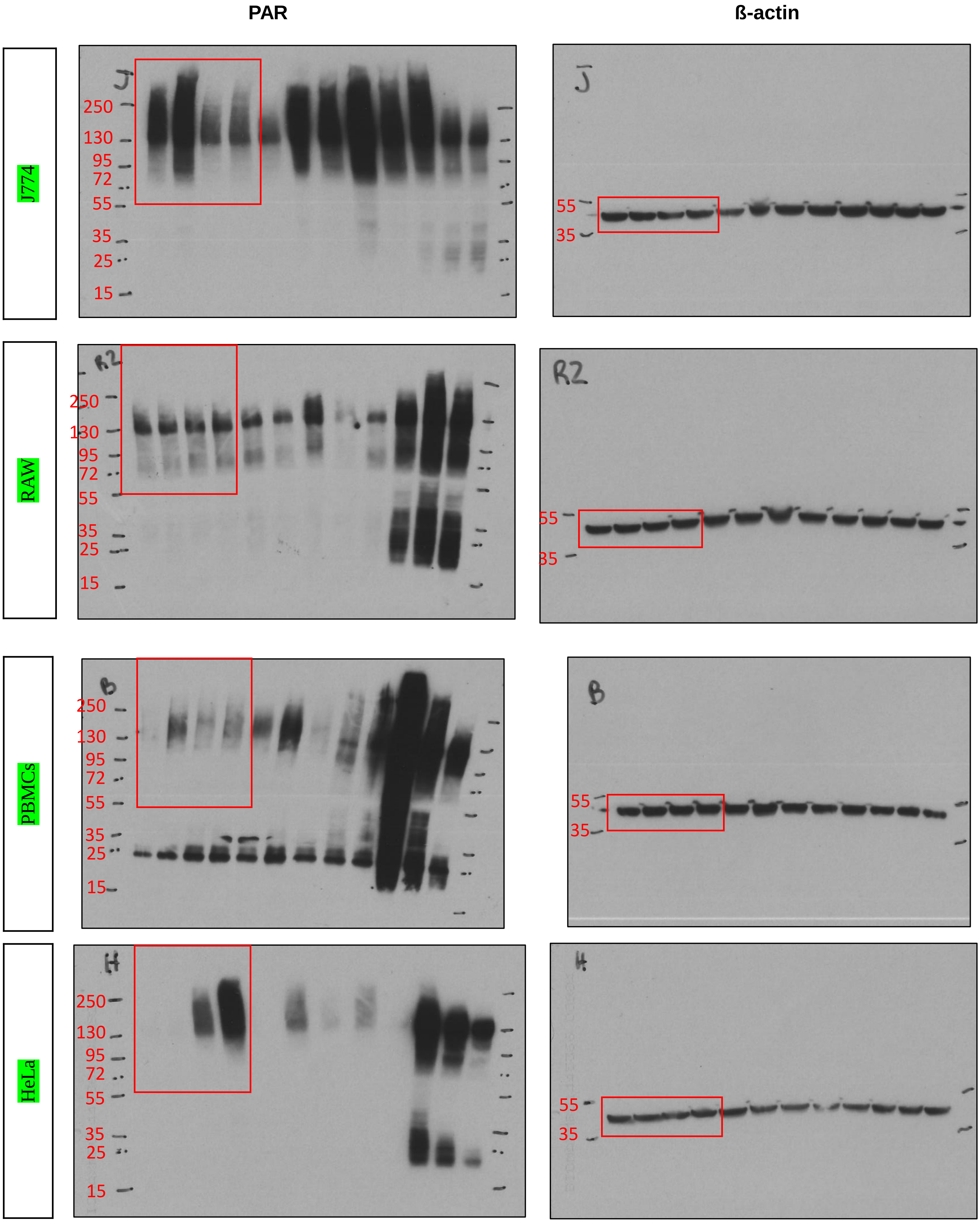

Supplementary Fig. 2b

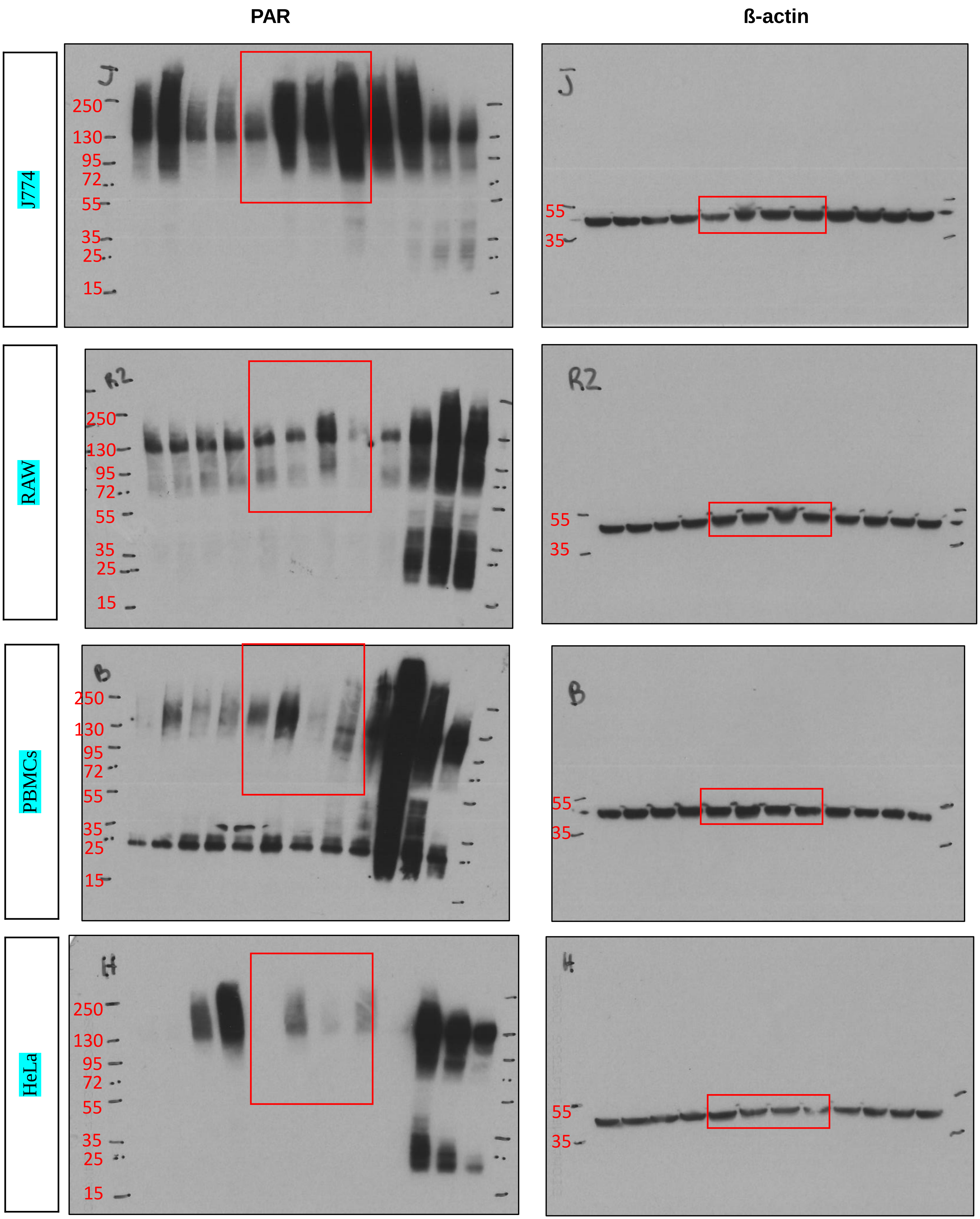

Supplementary Fig. 2c

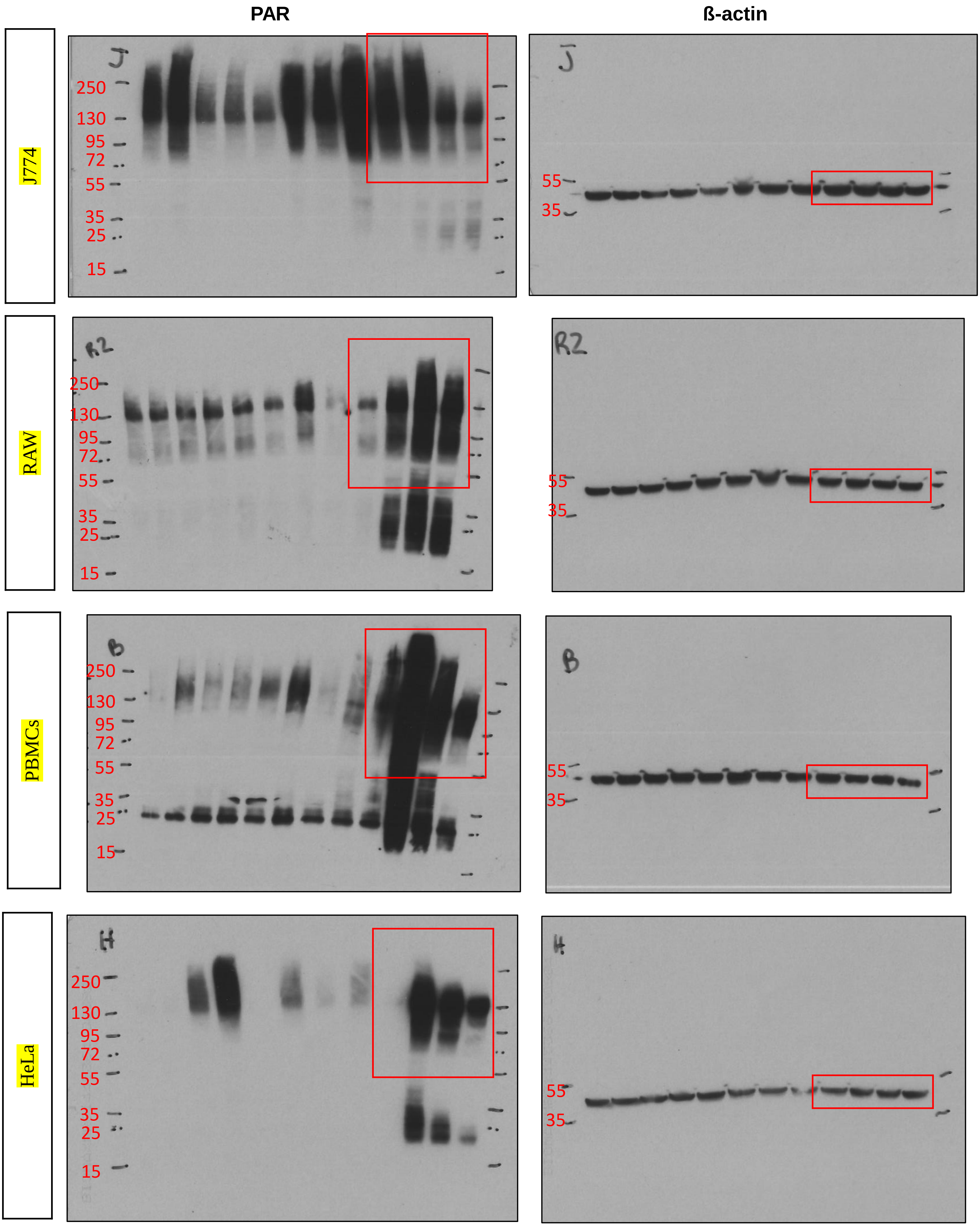

Supplementary Fig. 2d

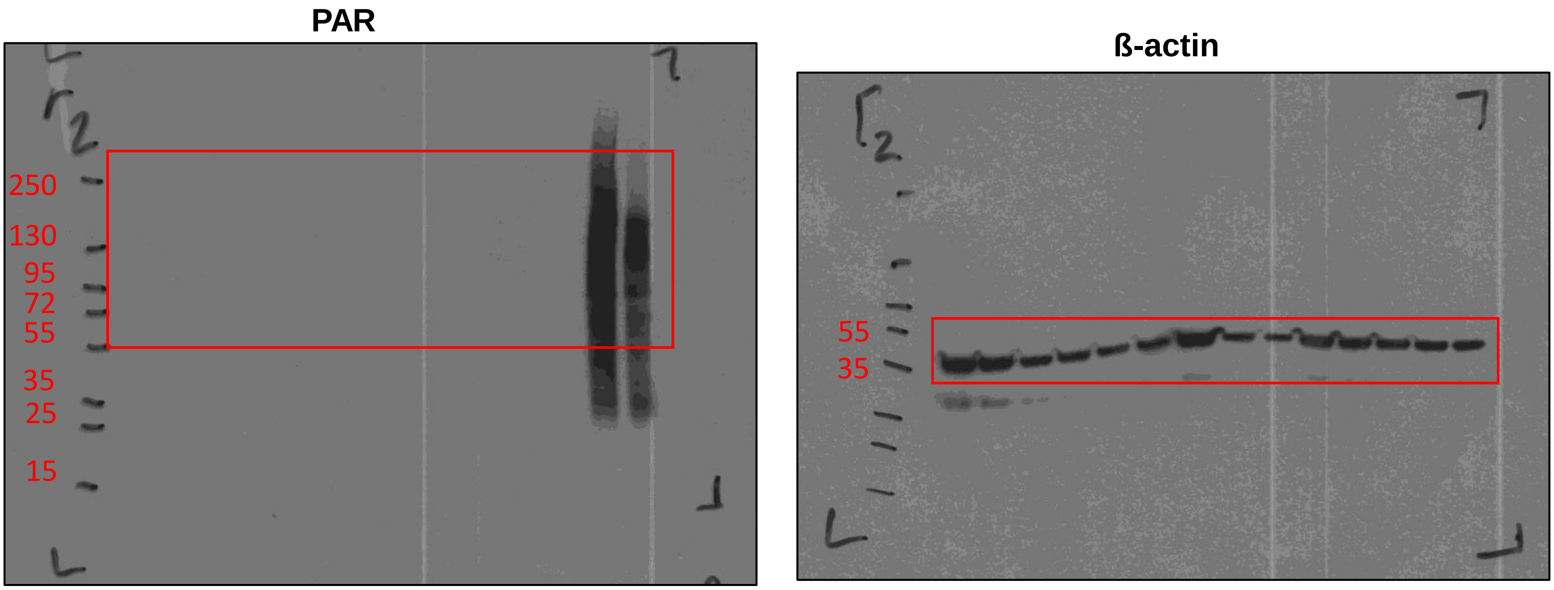

Supplementary Fig. 3a

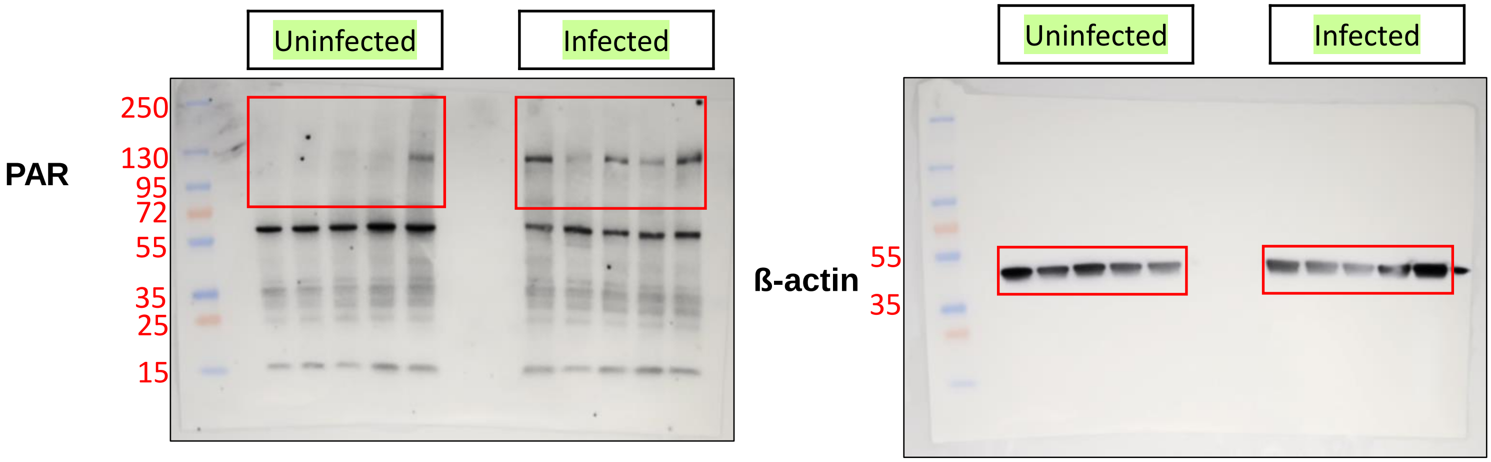

Supplement: Supplementary file 1 — Supplementary Information [file 41467_2023_43937_MOESM1_ESM.pdf]
