# Supplementary material for: Inhibition of host PARP1 contributes to the anti-inflammatory and antitubercular activity of pyrazinamide
Source: Nat Commun. 2023 Dec 9;14:8161. doi: 10.1038/s41467-023-43937-1 (PMC10710439; doi:10.1038/s41467-023-43937-1)
Supplement: Supplementary file 3 — Reporting Summary [file 41467_2023_43937_MOESM3_ESM.pdf]

nature portfolio

Corresponding author(s): William R. Bishai

Last updated by author(s): Nov 14, 2023

Reporting Summary

Nature Portfolio wishes to improve the reproducibility of the work that we publish. This form provides structure for consistency and transparency in reporting. For further information on Nature Portfolio policies, see our Editorial Policies and the Editorial Policy Checklist.

Statistics

For all statistical analyses, confirm that the following items are present in the figure legend, table legend, main text, or Methods section.

n/a	Confirmed	
☐	☒	The exact sample size (*n*) for each experimental group/condition, given as a discrete number and unit of measurement
☐	☒	A statement on whether measurements were taken from distinct samples or whether the same sample was measured repeatedly
☐	☒	The statistical test(s) used AND whether they are one- or two-sided<br>*Only common tests should be described solely by name; describe more complex techniques in the Methods section.*
☐	☒	A description of all covariates tested
☐	☒	A description of any assumptions or corrections, such as tests of normality and adjustment for multiple comparisons
☐	☒	A full description of the statistical parameters including central tendency (e.g. means) or other basic estimates (e.g. regression coefficient) AND variation (e.g. standard deviation) or associated estimates of uncertainty (e.g. confidence intervals)
☐	☒	For null hypothesis testing, the test statistic (e.g. *F*, *t*, *r*) with confidence intervals, effect sizes, degrees of freedom and *P* value noted<br>*Give P values as exact values whenever suitable.*
☒	☐	For Bayesian analysis, information on the choice of priors and Markov chain Monte Carlo settings
☒	☐	For hierarchical and complex designs, identification of the appropriate level for tests and full reporting of outcomes
☐	☒	Estimates of effect sizes (e.g. Cohen's *d*, Pearson's *r*), indicating how they were calculated

Our web collection on statistics for biologists contains articles on many of the points above.

Software and code

Policy information about availability of computer code

Data collection	For the thermal shift assay and quantitative real-time PCR, data was collected on a StepOnePlus Real-Time PCR System using the StepOne software v2.3 (Applied Biosystems). Primers were designed using the GenScript Real-time PCR Primer Design tool (TaqMan). Images of chemiluminescent Western blots were captured using the KwikQuant digital imager (Kindle Biosciences, LLC). Cytokine/chemokine concentrations were determined by Luminex multiplex bead assay on a Bio-Plex 200 platform (Bio-Rad). Histology and IHC slides were digitally scanned on an Aperio AT turbo scanner console version 102.0.7.5 (Leica Biosystems) and transferred using Concentriq for Research version 2.2.4 (Proscia Inc).
Data analysis	For the molecular docking studies, PZA was manually docked into the PARP1 crystal structure in COOT software, and figures were prepared using PyMOL Molecular Graphics System, Version 1.5.0.4 (Schrödinger, LLC). Melting temperatures and differential fluorescence values were calculated in GraphPad Prism version 5.01 for Windows (GraphPad). Quantitative real-time PCR data were analyzed using StepOne Software v2.3 (Applied Biosystems) and Microsoft Excel version 2310 (Microsoft Office Professional Plus 2019). Digital Western blot images were converted to black-and-white using Photoshop, and relative band intensities were quantified using ImageJ (version 1.52a). Histology slides were analyzed using Aerie ImageScope software (Leica Biosystems). IHC staining was analyzed using QuPath digital pathology image analysis software (total lung area channel: average channels, smoothing sigma 3, threshold 225; immuno-positive area channel: DAB, smoothing sigma 1, threshold 0.14). Statistical analyses were performed in Prism version 9.2.0 for Windows (GraphPad). All artwork used in this manuscript is original and was drawn using the vector graphics editor Inkscape for windows v. 0.92.4.

For manuscripts utilizing custom algorithms or software that are central to the research but not yet described in published literature, software must be made available to editors and reviewers. We strongly encourage code deposition in a community repository (e.g. GitHub). See the Nature Portfolio guidelines for submitting code & software for further information.

Data

Policy information about availability of data

All manuscripts must include a data availability statement. This statement should provide the following information, where applicable:

- Accession codes, unique identifiers, or web links for publicly available datasets
- A description of any restrictions on data availability
- For clinical datasets or third party data, please ensure that the statement adheres to our policy

Source data generated in this study are provided with this paper in the 'Source Data' file. A repository of the digitally scanned H&E-stained lung sections used for histology analysis is publicly available at https://tinyurl.com/4az6ueks. A repository of the digitally scanned IHC-stained lung sections is publicly available at https://tinyurl.com/3v384esc, and the decoded slide IDs are listed in the 'Source Data' file. Uncropped and unprocessed Western blots are supplied in the file 'Uncropped Western blots' (main display items) or at the end of the 'Supplementary Data' file (supplementary figures).

Research involving human participants, their data, or biological material

Policy information about studies with human participants or human data. See also policy information about sex, gender (identity/presentation), and sexual orientation and race, ethnicity and racism.

Reporting on sex and gender	n/a
Reporting on race, ethnicity, or other socially relevant groupings	n/a
Population characteristics	n/a
Recruitment	n/a
Ethics oversight	n/a

Note that full information on the approval of the study protocol must also be provided in the manuscript.

Field-specific reporting

Please select the one below that is the best fit for your research. If you are not sure, read the appropriate sections before making your selection.

☒ Life sciences ☐ Behavioural & social sciences ☐ Ecological, evolutionary & environmental sciences

For a reference copy of the document with all sections, see nature.com/documents/nr-reporting-summary-flat.pdf

Life sciences study design

All studies must disclose on these points even when the disclosure is negative.

Sample size	All sample sizes are provided in the figure legends. Appropriate sample sizes were determined empirically (i.e., in pilot studies intended to evaluate effect size of unknown factors) and guided by relevant published literature. No power calculations were performed to determine sample sizes. For PAR detection in mouse tissues, a minimum of 3 mice per group were used based on the robustness of PAR signals in preliminary studies. For bacterial enumeration and cytokine analysis, a minimum of 5 mice per group and time point were used based on published literature and preliminary TB infection studies. For bacterial enumeration in C3HeB/FeJ mice, a minimum of 8 mice per group and time point were used to account for the well-known heterogeneity in bacterial burden following aerosolized TB infection and the fact that approximately 20% of infected C3HeB/FeJ mice do not respond to PZA; both of these observations have been extensively described in the literature. For histology and inflammation scoring, 3 mice per group were deemed sufficient to capture differences in TB lung disease following 2 months of treatment with antibiotics (PZA, RIF) based on previous studies performed in the lab. One Tp-treated mouse in the histology study died prior to the completion of the study, reducing the sample size from 3 to 2 in that group.
Data exclusions	All data collected are reported in the 'Source Data' file, with any values excluded from analysis indicated. We defined outliers as any value that fell more than 1.96 standard deviations above or below the group mean; in some instances these outliers were excluded from analysis. For CFU analysis, we also excluded any sample for which an undiluted sample resulted in fewer than 5 colonies on a plate.
Replication	PZA inhibition of PARP1 activity in macrophages was repeated 7 independent times, and data from all studies are shown in this manuscript. All attempts resulted in comparable results. For all studies involving mouse models of TB infection, sufficient biological replicates to capture the natural variability and reproducibility of each parameter were included. Mouse studies were repeated twice with similar outcomes. PAR formation in TB-infected mouse lungs was assessed in at least 5 independent infections, with comparable results.
Randomization	After aerosol infection, animals were randomly assigned into the individual treatment groups. Bacterial colonies were counted blinded to experimental grouping and in a randomized order. Histopathology and IHC analyses were performed by specialists blinded to experimental design, and samples were provided coded and randomized to ensure consistent scoring. For cytokine/chemokine analysis, the plate setup was determined by a core facility technician blinded to experimental design and grouping; while samples from the same time point were grouped

together, the order on the plate was randomized. For PAR blots, representative samples were selected from the control and experimental groups at random, ensuring that different blots contained a random sampling of each group. In addition, the order in which groups were loaded on the gel was routinely varied to avoid "lane artifacts".

Blinding	During tissue collection, investigators were not blinded to experimental grouping to ensure that tissues were harvested in the order of predicted increasing bacterial burden (i.e., uninfected first, RIF + PZA-treated second, vehicle-treated last). Every animal was then assigned a unique numerical identifier that contained no information about the group it belonged to to ensure that all data would be analyzed blinded to experimental grouping. Bacterial colonies were counted blinded to experimental grouping and in a randomized order. Cytokine/chemokine, histology, IHC and qPCR analyses were performed by trained professionals blinded to experimental design and identity of samples. For Western blot analysis, investigators were aware of the identity of each sample so that the appropriate comparisons were run on the same gel (i.e., vehicle-treated vs. drug-treated).

Reporting for specific materials, systems and methods

We require information from authors about some types of materials, experimental systems and methods used in many studies. Here, indicate whether each material, system or method listed is relevant to your study. If you are not sure if a list item applies to your research, read the appropriate section before selecting a response.

Materials & experimental systems

n/a	Involved in the study
☐	☒ Antibodies
☐	☒ Eukaryotic cell lines
☒	☐ Palaeontology and archaeology
☐	☒ Animals and other organisms
☒	☐ Clinical data
☒	☐ Dual use research of concern
☒	☐ Plants

Methods

n/a	Involved in the study
☒	☐ ChIP-seq
☒	☐ Flow cytometry
☒	☐ MRI-based neuroimaging

Antibodies

Antibodies used	Human monoclonal anti-PAR antibodies (clones #19 and #21) used for PAR detection were custom-designed at Bio-Rad AbD Serotec GmbH, as described in PMID: 24987120. Dilution: 1:2,500 in 5% nonfat dry milk-TBST. The anti-PAR antibodies were generously provided by Valina Dawson.<br>Secondary antibody for PARP detection: HRP-conjugated goat anti-human IgG (Fab')2 (Abcam, cat. no. 87422). Dilution: 1:5,000 in 1% nonfat dry milk-TBST.<br>Loading control: HRP-conjugated mouse monoclonal anti-beta Actin antibody [AC-15] (Abcam, cat. no. ab49900). Dilution: 1:50,000 in 5% (w/v) BSA in TBST.
Validation	The monoclonal PAR antibody was generated against purified PAR (Trevigen) using HuCAL technology at AbD Serotec. All antibodies that detected free ADP-ribose were removed, and highly specific human monoclonal recombinant antibodies against target-bound PAR were generated (as described in PMID: 24987120). These antibodies were screened in human and mouse cells by the authors.<br>The secondary antibody for PAR detection (Abcam # ab87422) is a polyclonal antibody raised against the Fab and F(ab')2 portion of human IgG. Specificity was confirmed by IEP by the manufacturer. It can react with other human immunoglobulins through common light chain reactivity and will react with less than 1% of the Fc domain of human IgG.<br>The beta-actin antibody (Abcam # ab49900) has been extensively validated by the manufacturer and is widely used in the scientific literature. As stated by Abcam, The Life Science industry has been in the grips of a reproducibility crisis for a number of years. Abcam is leading the way in addressing this with our range of recombinant monoclonal antibodies and knockout edited cell lines for gold-standard validation.

Eukaryotic cell lines

Policy information about cell lines and Sex and Gender in Research

Cell line source(s)	THP-1 (ATCC TIB-202; human monocyte cell line derived from a male individual with acute monocytic leukemia), Raw 264.7 (ATCC TIB-71; a macrophage cell line established from a tumor in a male mouse induced with the Abelson murine leukemia virus), HeLa (ATCC CCL-2; a human epithelial cell line derived from a female individual with cervical carcinoma) and J774A.1 (ATCC TIB-67; a murine monocytic cell line isolated from the ascites of an adult, female mouse with reticulum cell sarcoma) were purchased from ATCC. Human peripheral mononuclear cells (PBMCs) from two healthy anonymous donors (sex unknown) were a generous gift from Dr. Andrea Cox (Johns Hopkins, Baltimore).
Authentication	None of the cell lines were authenticated.
Mycoplasma contamination	Cell lines were not tested for mycoplasma contamination.
Commonly misidentified lines (See ICLAC register)	No commonly misidentified cell lines were used in this study.

Animals and other research organisms

Policy information about studies involving animals; ARRIVE guidelines recommended for reporting animal research, and Sex and Gender in Research

Laboratory animals	This study utilized female C3Heb/FeJ (Jackson Laboratory, stock 658), male and female 129S1/SvlmJ (Jackson Laboratory, stock 002448), and male and female 129S-Parp1tm1Zqw (Jackson Laboratories, strain 002779) mice. 129S-Parp1tm1Zqw mice were bred in house; all other mice were purchased from Jackson Laboratories for each study. Mice were infected between 8 and 12 weeks of age. Mice were housed on a 12h:12h light:dark cycle with access to mouse chow and water ad libitum.
Wild animals	No wild animals were used in this study.
Reporting on sex	Infections in C3HeB/FeJ mice utilized only female mice, as has been the standard used by most labs for mouse models of TB infection. Experiments comparing effects in 129S1/SvlmJ and 129S-Parp1tm1Zqw used equal numbers of male and female mice. The parameters compared in this study did not differ between males and females, and data from male and female mice were combined and are presented as one group throughout the manuscript. All data is reported disaggregated for sex in the 'Source Data' file.
Field-collected samples	This study did not involve samples collected in the field.
Ethics oversight	All protocols for animal studies and breeding were approved by the Institutional Animal Care and Use Committee of the Johns Hopkins University School of Medicine.

Note that full information on the approval of the study protocol must also be provided in the manuscript.

Plants

Seed stocks	This study did not involve seed stocks or plants.
Novel plant genotypes	n/a
Authentication	n/a
