# Supplementary material for: Inhibition of host PARP1 contributes to the anti-inflammatory and antitubercular activity of pyrazinamide
Source: Nat Commun. 2023 Dec 9;14:8161. doi: 10.1038/s41467-023-43937-1 (PMC10710439; doi:10.1038/s41467-023-43937-1)

Inhibition of host PARP1 contributes to the anti-inflammatory and antitubercular activity of pyrazinamide

Stefanie Krug[1,2], Manish Gupta[1,2], Pankaj Kumar[1,2], Laine Feller[2], Elizabeth A. Ihms[1,2], Bong Gu Kang[3,4], Geetha Srikrishna[1,2], Ted M. Dawson[3,4,5,6], Valina L. Dawson[3,4,5,7], William R. Bishai[1,2]*

(NCOMMS-22-06952A)

UNCROPPED WESTERN BLOTS

Figure 2b

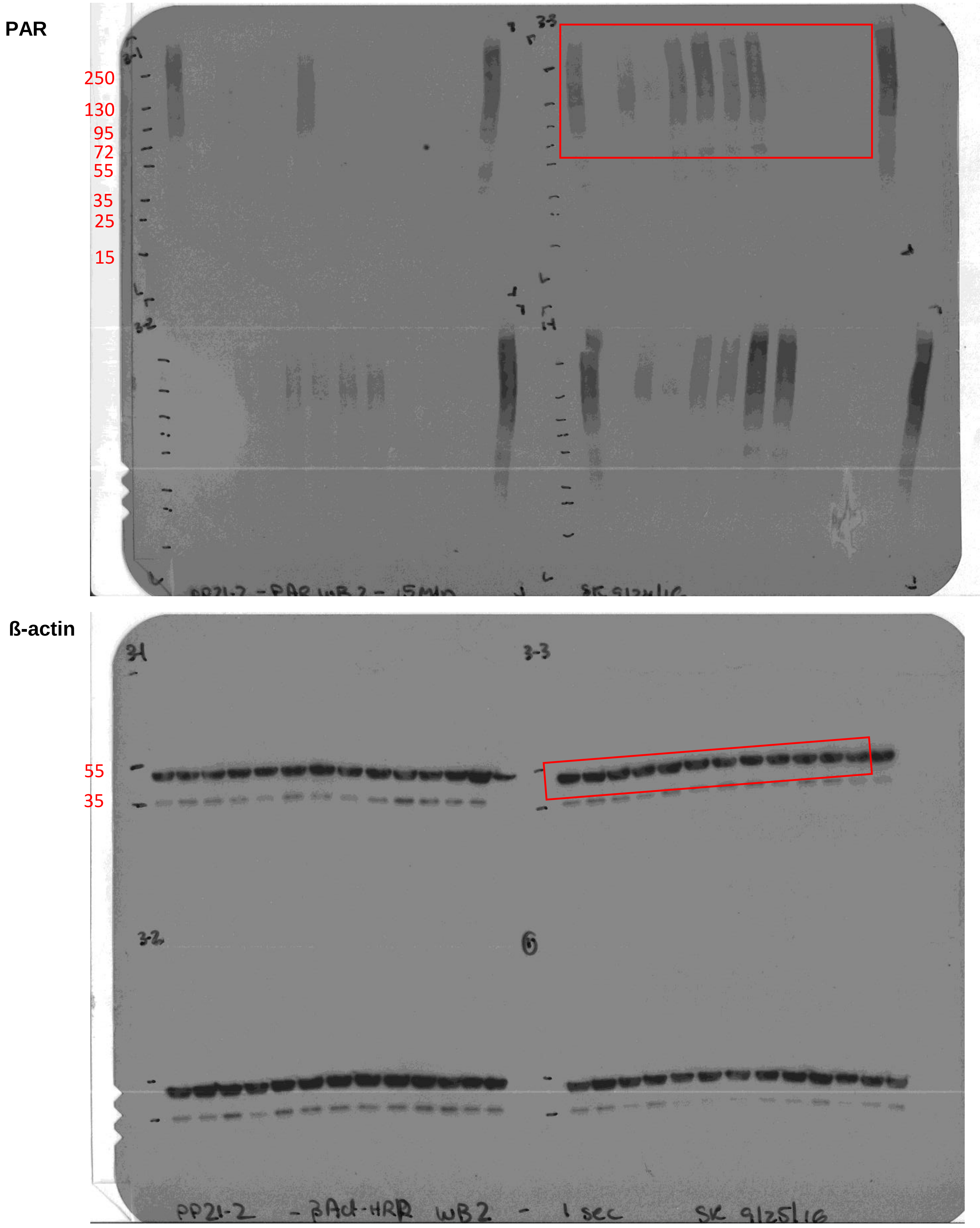
PAR
250
130
95
72
55
35
25
15
ß-actin
55
35
PP21-2 - βAct-HRP WB2 - 1 sec SK 9/25/16

Figure 3b – uncropped blots

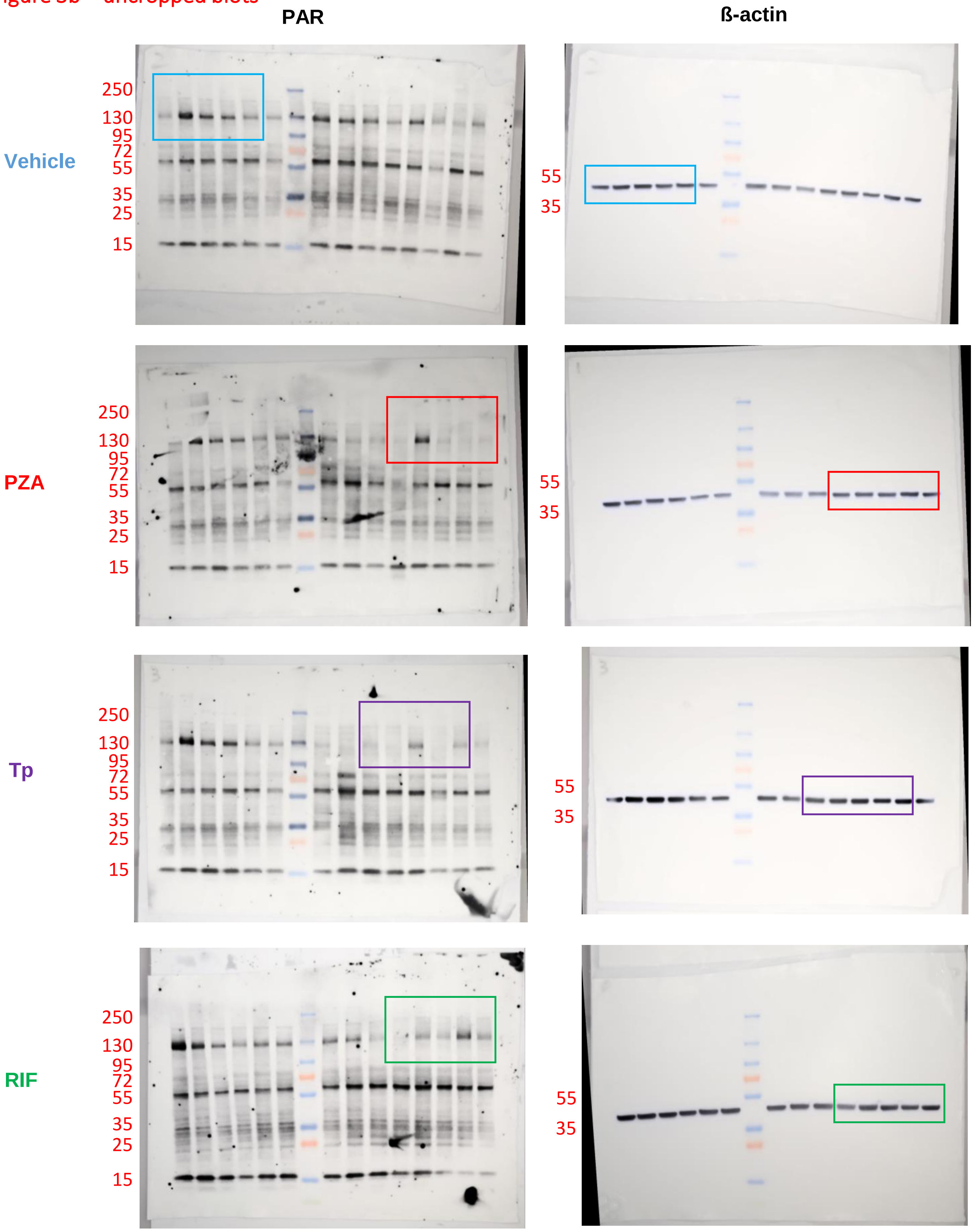

Figure 3b – unprocessed blots

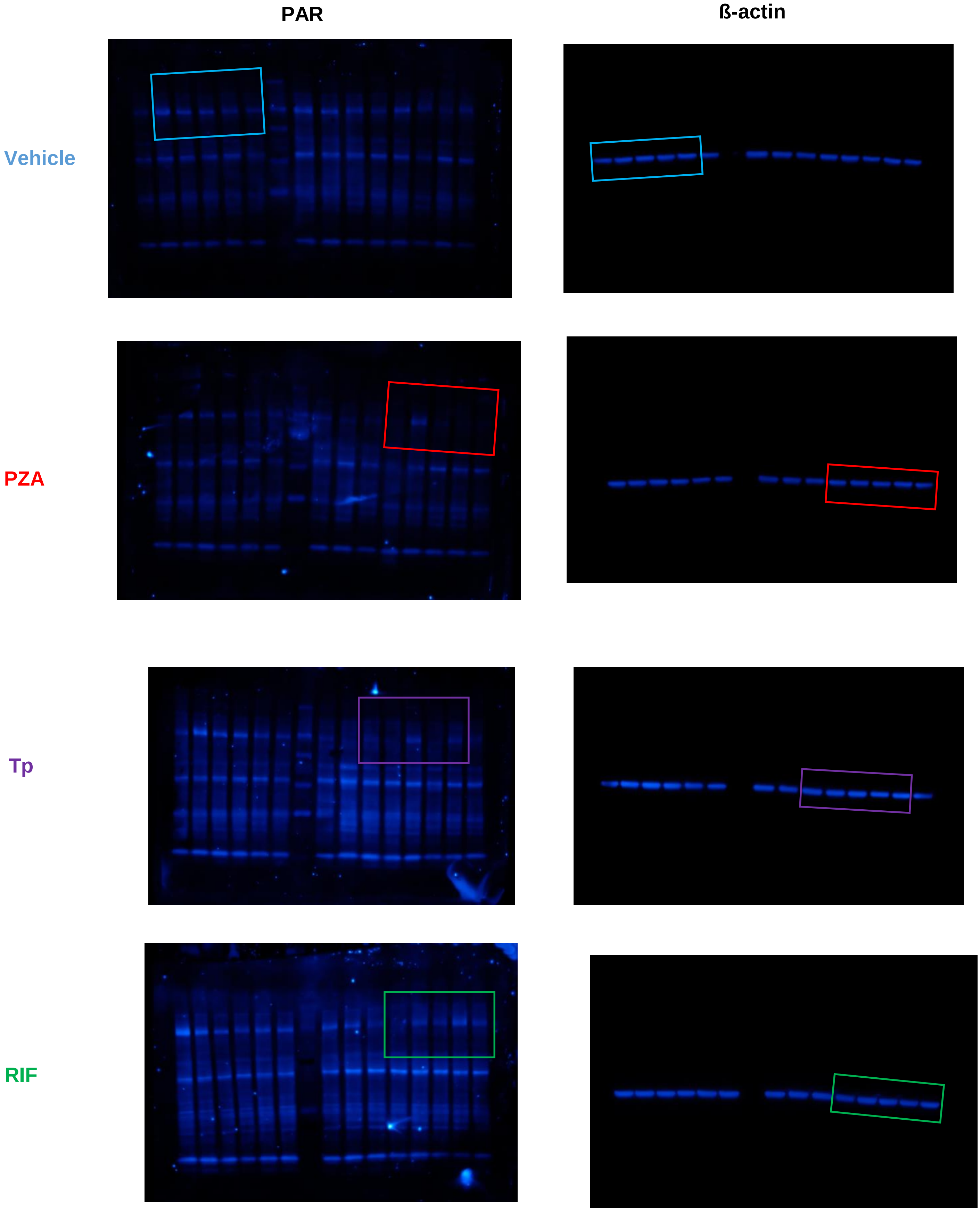

Supplement: Supplementary file 4 — Source Data [file 41467_2023_43937_MOESM4_ESM.zip › Uncropped Western Blots.pdf]
